# Comparison of Easydo Activator, ultrasonic and needle irrigation techniques on sealer penetration and smear layer removal in vitro

**DOI:** 10.1186/s12903-023-03833-y

**Published:** 2024-01-09

**Authors:** Shao-Hui Zhang, Zheng-Rong Gao, Ying-Hui Zhou, Li Tan, Yao Feng, Qin Ye, Jie Zhao, Ya-Qiong Zhao, Jing Hu, Yun Chen, Qiong Liu, Dusenge Marie Aimee, Yue Guo, Yun-Zhi Feng

**Affiliations:** 1 Hunan Provincial Clinical Research Center for Oral Diseases, Hunan Provincial Engineering Research Center of Digital Oral and Maxillofacial Defect Repair, Department of Stomatology, The Second Xiangya Hospital, Central South University, Changsha, Hunan 410011 China; 2 Department of Stomatology, Xiangyang Central Hospital, Affiliated Hospital of Hubei University of Arts and Science, Xiangyang, Hubei 441000 China; 3 Department of Ultrasound Diagnosis, The Second Xiangya Hospital, Central South University, Changsha, Hunan 410011 China; 4 National Clinical Research Center for Metabolic Diseases, Hunan Provincial Key Laboratory of Metabolic Bone Diseases, Department of Metabolism and Endocrinology, The Second Xiangya Hospital of Central South University, Changsha, Hunan 410011 China

**Keywords:** Irrigation, Root canal therapy, Sealer penetration, Smear layer removal, Easydo activator, Scanning electron microscopy, Confocal laser scanning microscopy

## Abstract

The effects of Easydo Activator (EA), a new sonic irrigation system, on sealer penetration at the root apex were compared to needle irrigation (NI) and passive ultrasonic irrigation (PUI) in this study. Forty-two single-rooted teeth were prepared and randomly divided into three groups (n = 14): group 1: NI; group 2: PUI; and group 3: EA. A solution of 3% sodium hypochlorite (NaOCl) was used for irrigation. Nine teeth in each group were filled with AH Plus sealer mixed with CY5 fluorescent dye and a single gutta-percha cone. The sealer penetration area, maximum penetration depth and percentage of sealer penetration at 5 mm and 1 mm from the apex were analyzed by confocal laser scanning microscopy (CLSM). The remaining 5 teeth in each group were subjected to test smear layer scores by scanning electron microscopy (SEM). The CLSM evaluation showed that increases in the area, depth and percentage of sealer penetration were detected at 1 and 5 mm from the root apex in the PUI group compared with the NI group, and greater increases were observed in the EA group (*P* < 0.05). The SEM experiment showed that the lowest scores for the smear layer and debris removal were achieved by the EA group when compared with the PUI and NI groups (*P* < 0.05). In conclusion, EA was superior to PUI and NI regarding sealer penetration at the root apex during endodontic treatment, and it could provide a new technical idea for clinical root canal therapy.

## Introduction

The 3-dimensional filling of the cleaned and shaped root canal system is crucial to the endodontic success [1, 2]. To enhance the effect of root canal filling in eliminating infection, sealers must penetrate into the dentinal tubules [3–5]. Sealers penetrated into the dentinal tubules can eliminate the way of bacteria entering the root canal and prevent reinfection [6]. At the same time, deep penetration of sealers can improve the preservation time of the filling materials by mechanical retention [7]. However, the residual smear layer produced by mechanical instrumentation can adhere to the surface of dentin and occlude the dentinal tubules [8, 9], thus preventing sealers from penetrating into the dentinal tubules and might promote the invasion of bacterial to the dentinal tubules [10]. Thus, numerous irrigation devices have been developed to remove the smear layer and improve canal cleanliness and filling [11–13].

Conventional needle irrigation (NI) is the most commonly used irrigation technique. However, the application of NI alone cannot ensure the efficacy of canal preparation. NI fails to deliver irrigation solutions 0–2 mm past the needle tip and into intricate areas of root canals, such as the apical third, where gas particles can become entrapped to produce a vapor lock effect [14, 15]. Besides, NI is operated manually, the flow rate is difficult to be accurately controlled, which will also affect the effect of root canal cleaning [16]. Therefore, passive ultrasonic irrigation (PUI) was invented and has proved to be more effective than NI in removing pulpal tissue remnants and smear layers in the apical third due to its powers of acoustic streaming and cavitation [17, 18]. However, the effect of removing pulpal tissue remnants and the smear layer using PUI still need to be improved. Because the effect of PUI appears to decrease with increasing depth of the root canal system [19, 20].

Sonic activation is another irrigation system that uses a mechanical vibration technique for root canal therapy. It is controversial whether this irrigation system can improve root canal cleaning ability and dentinal tubule penetration of sealers. In previous studies, sonically activated irrigation needles with the Vibringe System (Vibringe B. V. Corp, Amsterdam, Netherlands) were proved to lead to increased fluid velocity of irrigation, and it could can better remove debris at the apex because of its higher oscillation amplitude at the tip than at the attached end [21]. Moreover, a similar study reported by Aksel et al. showed that sonically activated NFX irrigation needles (Ultradent, South Jordan, UT, USA) using the Vibringe System (Vibringe B. V. Corp, Amsterdam, Netherlands) could better remove smear layer and debris covered or packed into the dentinal tubules, which could lead to better sealer penetration into the dentinal tubules at 1 and 5 mm from the apex [22]. However, other studies have found that the use of sonic activation with the Vibringe System (Vibringe B. V. Corp, Amsterdam, Netherlands) did not significantly improve sealer penetration compared with NI (Dentsply Rinn, Elgin, IL) [23]. Easydo Activator (EA; Easyinsmile (WEIXIAOMEICHI), Changsha, China) is a new cordless sonic activation device (Dimensions, 355 mm×135 mm×255 mm; Frequency,3 kHz; Power Input, 100 to 240 V to 50 Hz/60Hz) that uses highly flexible polyamide tips with three taper models (35/0.04, 25/0.04 and 15/0.02) to deliver irrigants (Chinese invention patent, patent NO. CN 201922435016.6). The highly flexible polyamide tips are soft and flexible and can avoid contact with the canal walls during irrigation, leading to less unintentional dentin removal and increasing sealer penetration. At the same time, the three-dimensional movement of the highly flexible polyamide tips allows EA to efficiently and promptly deliver irrigants into the root canals, particularly in the apical third of the root canal, to achieve prominent cleaning efficiency and improve the success rate of root canal treatment [24]. To the best of our knowledge, the effects of EA on sealer penetration have not been studied.

In this study, the effect of EA treatments on sealer penetration at the root apex was evaluated by combining confocal laser scanning microscopy (CLSM) and scanning electron microscopy (SEM) methods. The usage of these methods both allows for standard and reproducible three-dimensional imaging of the samples [25, 26] and provides a comprehensive and detailed analysis of the sealing interface [11, 27, 28]. The null hypothesis was that there would be no difference in sealer penetration among the three different irrigation techniques.

## Methods

### Sample size calculation

This study was approved by the Institutional Review Board of the Second Xiangya Hospital, Central South University (No. 2021031) and the methods were carried out in accordance with the Declaration of Helsinki (2008). Informed consent was obtained from all patients before sample collection. Sample size calculation was performed with using PASS software (ver. 15.0; NCSS Inc., Kaysville, UT, USA) using the following parameters: two-tailed 5% significance level (α = 0.05), 95% confidence interval, 85% statistical power (β = 0.15), and a 1:1 ratio of sample allocation in the experimental groups. The minimum sample size for CLSM analysis was calculated to be 9 in each group, while the minimum sample size for SEM analysis was 3 in each group. Thus, the sample size was determined to be 9 in each group for CLSM analysis and 5 in each group for SEM analysis, which was more than or equal to the minimum sample size.

### Tooth selection and preparation

Freshly extracted human mature mandibular premolars with a single root canal and no apical absorption were selected for this experiment. The teeth were extracted from young adult patients with orthodontic therapeutic indications. The single root canal was evaluated by digital radiographs in both the buccolingual and mesio-distal directions. Teeth subjected to restorative or endodontic treatment were excluded. Teeth were kept in 0.9% sodium chloride solution containing 0.02% sodium azide at 4 °C to prevent bacterial growth [29]. To standardize canal instrumentation, the crowns of teeth were removed, and the roots were set at 12 mm. The working length (WL) of the root canal was determined by subtracting 1 mm from the distance to the apical foramen by 10 K file. After the apical foramen was filled with light-cured composite resin (Z-100, 3 M, Saint Paul, MN, USA), anatomical diameter was determined by a single operator with K files No. 10, 15 and 20 (Dentsply Maillefer, Ballaigues, Switzerland) in ascending order. The root canal’s diameter of Forty two teeth was pre-operatively standardized with an initial apical diameter correspondent to a size 10 K-file and prepared by using the X-Taper Universal files (Easyinsmile, Staten Island, NY, USA) to a master apical file size to 30/0.06 [30]. During canal preparation, a 30-gauge side-vented needle (Dentsply Tulsa Dental, Tulsa, OK, USA) filled with 2 mL 3.0% sodium hypochlorite (NaOCl) solution was used to irrigate the root canals between each file change.

### Final irrigation procedures

After completion of the chemomechanical preparation, all specimens were randomly divided into a control group and two experimental groups (n = 42): group 1: NI (n = 14); group 2: PUI (n = 14); and group 3: EA (n = 14). In group 1, each root canal was irrigated with a continuous flow of 3% sodium hypochlorite (NaOCl) (1.5 mL) for 45 s within 1 mm of the WL using a disposable syringe and a 30-gauge side-vented needle (Dentsply Tulsa Dental, Tulsa, OK, USA). Then, 2 mL of sterilized water were irrigated into the root canal using the same method. In group 2, passive ultrasonic irrigation (PUI, Easyinsmile, Staten Island, NY, USA) with an ultrasonic tip (DTE Endo File; EMS, Nyon, Switzerland) 25/0.04 was placed 1 mm from the WL at a frequency of 28 ± 3 kHz. An intermittent flush technique was used for the whole irrigation process with a total irrigation volume of 1.5 mL of 3% NaOCl for 3 cycles of 15 s. In the intermittent flush technique, the irrigant in a syringe is injected into the root canal and replenished after each ultrasonic activation cycle several times. In group 3, EA (Easyinsmile (WEIXIAOMEICHI), Changsha, China) with a 25/0.04 EA tip was placed 1 mm short of the WL at a frequency of 3 kHz (2 gear powers) to deliver 3% NaOCl (1.5 mL) for 45 s. After each respective irrigant, all root canals were dried with paper points.

### CLSM preparation and analysis

Nine teeth in each group were sealed with AH Plus sealer (Dentsply, DeTrey, Konstanz, Germany) mixed with CY5 fluorescent dye (0.1%; 0.001% per 1 g sealer; Bereket Chemical Industry, Istanbul, Turkey) and a single gutta-percha cone (ProTaper Universal F3, Dentsply Maillefer, Ballaigues, Switzerland). A #25.02 Lentulo spiral (Dentsply Maillefer, Ballaigues, Switzerland) attached to a handpiece at 20,000 rpm was inserted into the canal for 5 s to allow the sealer to be placed 1 mm short of the WL. After root filling, the coronal access was filled with temporary filling material (Cavit G, 3 M; ESPE, St. Paul, MN, USA), and then specimens were stored in an incubator at 100% humidity and 37 °C for 1 week for the next CLSM analysis.

Then, each sample was sectioned perpendicular to the long axis using a precision saw (EXAKT 300 CP; EXAKT, Norderstedt, Germany). Two slices were obtained from each tooth at depths of 5 mm and 1 mm and approximately 1 ± 0.1 mm in thickness. The sections were polished with an EXAKT grinder (EXAKT 400 CS; EXAKT, Norderstedt, Germany). The samples were then mounted onto glass slides and examined with confocal laser scanning microscopy (LSM800; ZEISS, Jena, Germany) at ×10 magnification with a wavelength of 560–600 nm.

The results of CLSM were analyzed by ImageJ software (ImageJ 2×, Rawak Software Inc., Stuttgart, Germany). The sealer penetration area was measured in micrometers and converted to square millimeters for statistical analysis. The maximum penetration depth was measured from the canal wall to the point of maximum sealer penetration. To determine the percentage of sealer penetration, the circumference of the root canal wall was measured, and areas along the canal walls into which the sealer penetrated the dentinal tubules at any distance were calculated. Then the outlined areas were divided by the canal circumference to calculate the percentage of sealer penetration.

### SEM preparation and analysis

To describe the effect of the final irrigation protocol on the removal of debris and smear layer from root canal walls, five samples without root canal filling in each group were observed by scanning electron microscopy (SEM). Every sample was separated longitudinally in the buccolingual direction using a bone hammer and bone chisels as reported by Shu Wan [31]. Horizontal marks were made at the apical sections on the cut/split dentin surface outside the root canal using a sharp scalpel. The samples were dried, mounted on metallic stubs, and examined under SEM (JSM-IT100; Jeol, Tokyo, Japan) at 10 kV. Photomicrographs at the apical thirds of each specimen were obtained at ×1000 and ×2000. The images at ×1000 were used for smear layer evaluation. The images were evaluated by two practitioners who were blinded to group assignment and final irrigation procedures. Kappa value was analyzed to evaluate inter-rater reliability between two practitioners, with a kappa coefficient exceeding 0.75 considered indicative of excellent or good agreement according to the 2008 guidelines for interpreting kappa values [32]. In our study, the kappa value was 0.820. The analysis was performed according to the four-level scoring system of Akyuz Ekim et al. [33]; Score 1: no smear layer or debris evidence on the dentinal tubules; Score 2: a few regions of the dentinal tubules covered with a smear layer and debris, with most tubules cleaned and opened; Score 3: most regions of the dentinal tubules covered with a smear layer and debris, with a few tubules cleaned and opened; and Score 4: the dentinal tubules completely covered with smear layer and debris.

### Statistical analysis

All of the data were analyzed using SPSS software (SPSS Statistics, version 23.0; SPSS Inc., IBM, Armonk, NY, USA). The CLSM data were calculated and expressed as the mean, median, standard deviation (SD), minimum, and maximum and evaluated using analysis of variance (ANOVA) with least significance difference (LSD) tests. For SEM scoring process, practitioners were previously calibrated for the scoring system to ensure interexaminer agreement. After achieving a good level of agreement (kappa ≥ 0.75), the practitioners scored the images independently. The kappa value in this study was 0.821. The SEM data were compared statistically by using the Kruskal–Wallis nonparametric analysis of variance. Mann–Whitney U-test was used for post hoc comparisons. A *P* value of 0.05 was considered statistically significant. GraphPad Prism software (GPW5-384305-RAG-5235, version 5.01; GraphPad Software Inc., San Diego, CA, USA) was used to draw diagrams.

## Results

### CLSM analysis

**Fig. 1:**
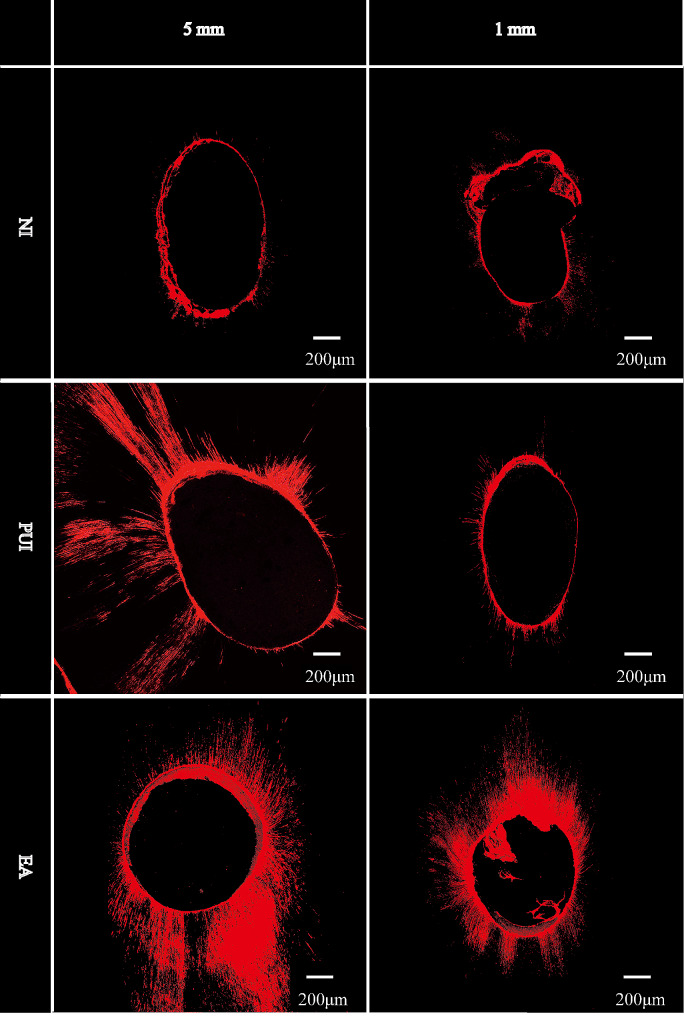
Representative CLSM images of each group at both 5 mm and 1 mm from the apex. CLSM, confocal laser scanning microscopy; NI, needle irrigation; PUI, passive ultrasonic irrigation; EA, Easydo Activator

**Fig. 2:**
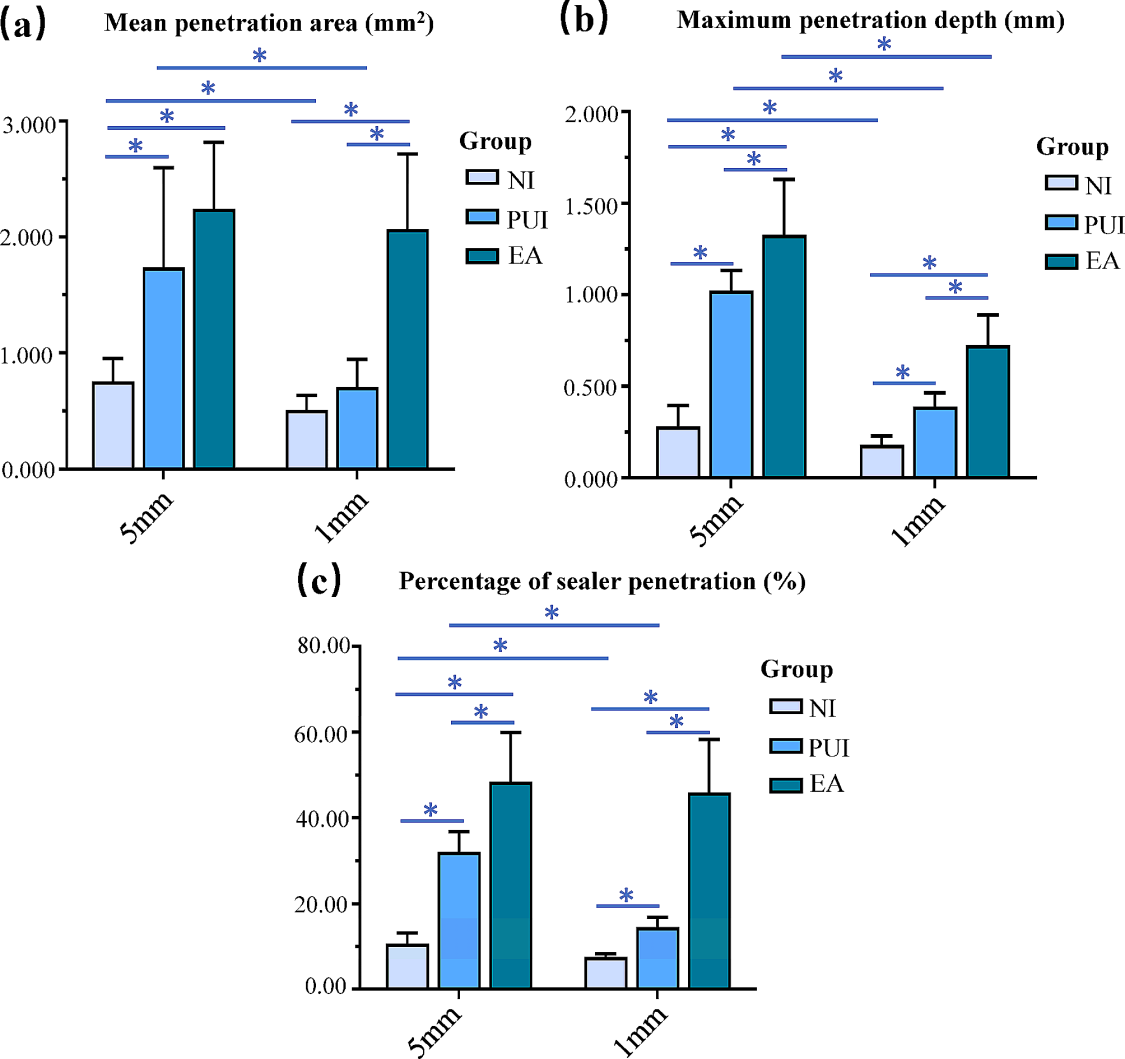
Column diagrams showing the mean ± SD of the mean penetration area (**a**), maximum penetration depth (**b**) and percentage of sealer penetration (**c**) for test groups at the levels of 5 mm and 1 mm from the apex. (^*^) indicates a significant difference at the 5% significance level (*P* < 0.05). NI, needle irrigation; PUI, passive ultrasonic irrigation; EA, Easydo Activator

Figure 1 shows representative CLSM images of each group at both 5 mm and 1 mm from the apex. The sealer penetration area (mm^2^), maximum penetration depth (mm) and percentage of sealer penetration (%) of each group at 5 mm and 1 mm from the apex are summarized in Tables 1 and 2, showing the mean, median, standard deviation, minimum, and maximum. The mean and SD of these results are shown in column diagrams in Fig. 2(a)–(c).

**Table 1:** Sealer penetration area (mm^2^), depth (mm), and percentage (%) of the test groups at 5 mm from the apex

Group	5 mm	No.	Mean	Median	Standard deviation	Minimum	Maximum
Group 1 Needle Irrigation	Area^†^	9	0.757	0.666	0.194	0.558	1.071
	Depth^†^	9	0.282	0.278	1.135	0.131	0.444
	Percentage^†^	9	10.75	10.92	2.420	7.740	14.37
Group 2 Passive ultrasonic irrigation	Area^‡^	9	1.738	1.638	0.861	0.594	3.348
	Depth^‡^	9	1.024	1.035	0.108	0.780	1.160
	Percentage^‡^	9	32.14	30.79	4.700	25.44	39.19
Group 3 Easydo Activator	Area^‡^	9	2.243	2.079	0.574	1.629	3.105
	Depth^§^	9	1.327	1.400	0.303	0.846	1.680
	Percentage^§^	9	48.51	44.35	11.45	34.39	65.63

Different superscript symbols indicate a significant difference at the 5% significance level (*P* < 0.05). (Data with the same superscript are not significantly different)

**Table 2:** Sealer penetration area (mm^2^), depth (mm), and percentage (%) of the test groups at 1 mm from the apex

Group	1 mm	No.	Mean	Median	Standard deviation	Minimum	Maximum
Group 1 Needle Irrigation	Area^†^	9	0.511	0.468	0.122	0.396	0.792
	Depth^†^	9	0.182	0.191	0.047	0.094	0.238
	Percentage^†^	9	7.640	7.540	0.630	6.790	8.440
Group 2 Passive ultrasonic irrigation	Area^†^	9	0.707	0.630	0.235	0.432	1.188
	Depth^‡^	9	0.391	0.354	0.073	0.297	0.532
	Percentage^‡^	9	14.57	15.12	2.330	10.62	17.35
Group 3 Easydo Activator	Area^§^	9	2.069	1.890	0.650	1.485	3.519
	Depth^§^	9	0.726	0.659	0.163	0.574	1.079
	Percentage^§^	9	45.94	45.73	12.37	34.15	74.33

Different superscript symbols indicate a significant difference at the 5% significance level (*P* < 0.05). (Data with the same superscript are not significantly different)

#### Mean penetration area

The sealer penetration areas at 5 mm from the apex in the NI and PUI groups were larger than that at 1 mm at the root canal level (*P* < 0.05), whereas no significant difference was observed in the EA group. EA and PUI increased the sealer penetration area more than NI at the 5 mm level (*P* < 0.05). There was no significant difference between the NI and PUI groups at 1 mm from the apex regarding the penetration area (*P* > 0.05). Activation with the EA instrument (2.069 ± 0.650 mm^2^) promoted more sealer penetration area at 1-mm root level compared to the other groups (*P* < 0.05).

#### Maximum penetration depth

The sealer penetration depth from the apical 5 mm was greater than that from the apical 1 mm in each group (*P* < 0.05). There were great differences in sealer penetration depth among different groups. The EA group (1.327 ± 0.303 mm at 5 mm, 0.726 ± 0.163 mm at 1 mm) was better than the PUI group (1.024 ± 0.108 mm at 5 mm, 0.391 ± 0.073 mm at 1 mm), and the PUI group was better than the NI group (*P* < 0.05).

#### Percentage of sealer penetration

The difference in the percentage of sealer penetration between 5 mm and 1 mm from the apex was not statistically significant in the EA group (48.51 ± 11.45% at 5 mm, 45.94 ± 12.37% at 1 mm; *P* ˃ 0.05), while the NI group and PUI group had a higher percentage of sealer penetration at the 5-mm level (10.75 ± 2.42% in NI, 32.14 ± 4.70% in PUI) than that in the 1-mm level (7.64 ± 0.63% in NI,14.57 ± 2.33% in PUI; *P* < 0.05). Large differences were found in the percentage of sealer penetration among the groups using different irrigating instruments (*P* < 0.05). The sealer infiltration percentage was significantly increased by EA compared with PUI and NI (*P* < 0.05).

### SEM analysis

**Fig. 3:**
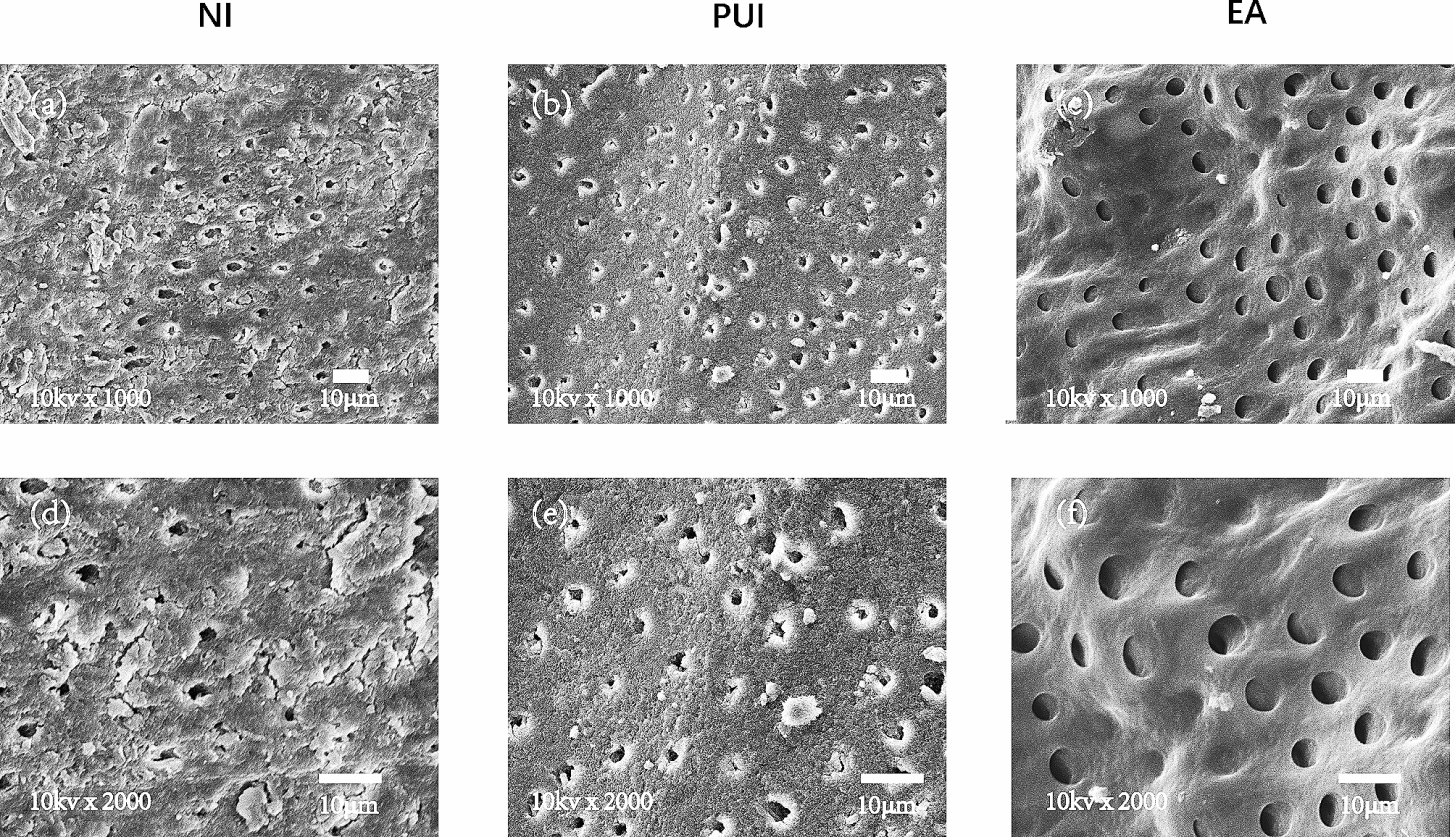
Representative SEM images of ×1000 and ×2000 in the apical thirds in the (**a**, **d**) NI group, (**b**, **e**) PUI group, and (**c**, **f**) EA group. SEM, scanning electron microscopy; NI, needle irrigation; PUI, passive ultrasonic irrigation; EA, Easydo Activator

Figure 3 shows representative SEM images at ×1000 and ×2000 of each group from the apex. The EA group (Fig. 3c, f) presented a smaller smear layer and debris covering the surface of dentinal tubules than the PUI group (Fig. 3b, e), and PUI (Fig. 3b, e) presented less smear layer and debris covering the surface of the dentinal tubules than NI (Fig. 3a, d). The results of the evaluated smear layer scores are summarized in Table 3, showing the mean and standard deviation. The smear layer score of the EA group (1.100 ± 0.316) was lower than that of the PUI group (2.500 ± 0.527; *P* < 0.05), and the smear layer score of the PUI group was lower than that of the NI group (3.700 ± 0.483; *P* < 0.05).

**Table 3:** Smear layer scores of the test groups at the apical third of the root canal

Group	No.	Mean	Standard deviation
Group 1 Needle Irrigation	5	3.700^†^	0.483
Group2 Passive ultrasonic irrigation	5	2.500^‡^	0.527
Group 3 Easydo Activator	5	1.100^§^	0.316

Different symbols indicate a significant difference at the 5% significance level (*P* < 0.05). (Data with the same superscript are not significantly different)

## Discussion

Removal of the smear layer from the canal walls during instrumentation allows for access of endodontic irrigants and sealers into the dentinal tubules [34, 35]. The EA instrument used in our study is a new cordless sonic activation device that uses highly flexible polyamide tips with three taper models (35/0.04, 25/0.04 and 15/0.02), and it is soft and flexible so that it can efficiently and promptly irrigate the root canals with less contact with the canal walls. Nonetheless, further research is warranted to provide a more detailed understanding of the cleaning efficiency of EA tips at the apex. The aim of this in vitro study was to evaluate the effects of different irrigation systems on AH Plus sealer penetration into the dentinal tubules by CLSM. Then, the removal of the smear layer was observed by SEM to confirm these findings. The present study data rejected the null hypothesis that there would be no differences in sealer penetration among the three different irrigation techniques.

The CLSM evaluation showed that, at 5 mm from the apex, EA and PUI exhibited greater penetration area, penetration depth and percentage of sealer penetration than NI (*P* < 0.05), in agreement with previous research by de Gregorio et al., who found that sonic activation devices (EndoActivator) and PUI have better sealer penetration than NI in the apical third (at 4.5 mm from WL) [36]. It has been reported that the main reason why EA and PUI have better sealer penetration than NI is that they increase the irrigant flow rate using different oscillation patterns [37, 38], which can better reduce vapor lock in the apical third of the root canal during irrigation [14, 39].

Moreover, markedly more sealer penetration was found at the level of 1 mm for EA than for PUI and NI (*P* < 0.05) in the evaluation of CLSM In addition, sealer penetration was decreased in the NI and PUI groups at 1 mm from the apex compared with that at 5 mm from the apex, whereas sealer penetration was not significantly decreased in the EA group (except for the penetration depth). In our study, EA uses a mechanical vibration technique that works at a frequency of 3 kHz and an amplitude of 150 µm, while the ultrasonic tip in PUI has a high frequency of approximately 28–32 kHz and amplitude of 28 µm [40]. Previous studies have shown that, in the apical third of the root canal, the oscillation amplitude could have a greater effect on the penetration of sealers and irrigants than the oscillation frequency [14, 40]. Therefore, we believe that one reason why EA has better sealer penetration at 1 mm from the apex than PUI is that the oscillation amplitude of EA is higher than that of PUI, even if the oscillation frequency of EA is lower than that of PUI. Another reason for markedly more sealer penetration at 1 mm from the apex found with EA than with PUI might be that, during irrigation, the amplitude of EA did not obviously change because the vibratory flexible polyamide tip is soft and flexible and has less contact with the root canal walls. In contrast, the ultrasonic tip of PUI is made of a rigid metal and can easily contact the canal walls when oscillating, which might sharply reduce the amplitude of the tip during irrigation and lead to production of smear layer by cutting the root canal dentin wall. The produced smear layer can act as a barrier, resulting in reduced sealer penetration [41, 42]. The higher and nonweakened oscillation amplitude in EA produces a higher irrigant flow rate, which has been reported to eliminate vapor lock and enhance sealer penetration [14].

Although the sonic activation device has a higher oscillation amplitude, it is puzzling that some studies have shown that sonic activation devices did not significantly improve sealer penetration at the root apex compared with NI irrigation and PUI [23, 43]. This different might be due to lower acoustic streaming generated by the sonic activation device with a small preparation taper size. Acoustic streaming is a very important factor for increasing the penetration of irrigants or sealers into dentinal tubules at the root apex [44]. If the preparation taper size is too small, the ability of the sonic activation device to generate acoustic streaming will be weaker because the capability of this device to generate acoustic streaming is based on the wide displacement amplitude of its tip, thus leading to a reduction in the penetration of irrigants or sealers into the dentinal tubules. In contrast, the acoustic streaming activated by PUI is minimally affected by the preparation taper size because PUI mainly activates acoustic streaming through a high vibration frequency rather than relying on a wide displacement amplitude [45]. The preparation taper size in our study (30/0.06) was larger than that used in the previous study (30/0.04) [45], which might be one reason why the EA group had more sealer penetration than the PUI group at the root apex. In addition, according to our study, a 30/0.06 taper might be sufficient for EA to generate sufficient acoustic streaming to result in better sealer penetration at the root apex.

Consistent with a previous study, SEM experiments further verified that PUI had greater ability to remove the smear layer than NI in the apical third because its high driving frequency of ultrasound (30 kHz) can lead to a high flow velocity of irrigant, resulting in more effective delivery of irrigant to the apical third of the root canal [21, 39]. Moreover, it was found that the smear layer and debris could be more effectively removed by EA compared to PUI and NI. This finding is not surprising because the tip of the EA had a higher oscillation amplitude, which could increase the flow velocity of the irrigant, thus achieving better removal of the smear layer in the apical third than PUI [42]. In addition, the sonic tip made of flexible polyamide could prevent the root canal dentin wall from cutting, resulting in less smear layer being produced in the canal dentin wall [46]. Since the residual smear layer produced by mechanical instrumentation can act as a barrier to decrease sealer penetration [8–10], the SEM finding that EA can effectively remove the smear layer was consistent with the CLSM findings.

This study still has some limitations. Firstly, the effects of different irrigation systems on sealer penetration and smear layer were studied in extracted teeth. In order to standardize canal instrumentation and set the roots at a uniform working length, similar to previous studies [30, 47–50], the crowns of extracted teeth were removed. However, this may lead to an incomplete extrapolation of the results to the clinic because the crown was removed. Therefore, this suggests that in the future, more in vivo studies may be needed to further evaluate the effects of different irrigation systems on root canal cleaning efficiency, in order to better apply the findings to clinical practice.

Additionally, in clinical practice, combining 17% EDTA with NaOCl as the irrigation solution is another common irrigation method, whereas we used only 3% NaOCl. Although this decision was based on the same considerations as those of Haupt, Urban K, and others, aiming to better assess the impact of different irrigation methods on enhancing root canal cleaning efficiency while excluding the influence of EDTA [11, 41]. It also suggests that we should interpret the results of this study with caution when extrapolating the findings to clinical outcomes using different irrigation solutions.

In clinical practice, the sealer penetration and smear layer removal in the apical third need to be improved, which is the key to increase the efficiency of cleaning and disinfection [51, 52]. The present study showed that EA was superior to PUI and NI in sealer penetration and smear layer removal at the apical third of the root canal. This finding suggests that EA is a promising irrigation device that can achieve noticeably superior cleaning and disinfection effects in the process of root canal irrigation. Therefore, the clinical application value of EA in root canal irrigation is worthy of further study.

## Data Availability

The datasets generated during and/or analyzed during the current study are available from the corresponding author on reasonable request.
